# Association of mental demands in the workplace with cognitive function in older adults at increased risk for dementia

**DOI:** 10.1186/s12877-021-02653-5

**Published:** 2021-12-10

**Authors:** Andrea E. Zülke, Melanie Luppa, Susanne Röhr, Marina Weißenborn, Alexander Bauer, Franziska-Antonia Zora Samos, Flora Kühne, Isabel Zöllinger, Juliane Döhring, Christian Brettschneider, Anke Oey, David Czock, Thomas Frese, Jochen Gensichen, Walter E. Haefeli, Wolfgang Hoffmann, Hanna Kaduszkiewicz, Hans-Helmut König, Jochen René Thyrian, Birgitt Wiese, Steffi G. Riedel-Heller

**Affiliations:** 1 Institute of Social Medicine, Occupational Health and Public Health (ISAP), Medical Faculty, University of Leipzig, 04103 Leipzig, Germany; 2 Global Brain Health Institute (GBHI), Trinity College Dublin, D02 PN40, Dublin, Ireland; 3 Department of Clinical Pharmacology and Pharmacoepidemiology, Heidelberg University Hospital, 69120 Heidelberg, Germany; 4 Institute of General Practice and Family Medicine, Martin-Luther-University Halle-Wittenberg, 06112 Halle (Saale), Germany; 5 Institute of General Practice/Family Medicine, University Hospital of LMU Munich, 80336 Munich, Germany; 6 Institute of General Practice, University of Kiel, 24105 Kiel, Germany; 7 Department of Health Economics and Health Service Research, University Medical Center Hamburg-Eppendorf, 20246 Hamburg, Germany; 8 Institute for General Practice, Work Group Medical Statistics and IT-Infrastructure, Hannover Medical School, 30625 Hannover, Germany; 9 Deutsches Zentrum für Neurodegenerative Erkrankungen (DZNE), site Rostock/ Greifswald, 17489 Greifswald, Germany; 10 Institute of Community Medicine, Dept. of Epidemiology of Health Care and Community Health, University Medicine Greifswald, 17487 Greifswald, Germany

**Keywords:** Mental demands, Cognition, Cognitive decline, Dementia, Risk factors, Workplace, Occupation

## Abstract

**Objectives:**

Growing evidence suggests a protective effect of high mental demands at work on cognitive function in later life. However, evidence on corresponding associations in older adults at increased risk for dementia is currently lacking. This study investigates the association between mental demands at work and cognitive functioning in the population of the *AgeWell.de*-trial.

**Methods:**

Cross-sectional investigation of the association between global cognitive functioning (Montreal Cognitive Assessment) and mental demands at work in older individuals at increased risk for dementia (Cardiovascular Risk Factors, Aging, and Incidence of Dementia (CAIDE)score ≥ 9; *n* = 941, age: 60–77 years). Occupational information was matched to Occupational Information Network (O*NET)-descriptors. Associations between cognitive function and O*NET-indices *executive, verbal* and *novelty* were investigated using generalized linear models.

**Results:**

Higher values of index *verbal* (b = .69, *p* = .002) were associated with better cognitive function when adjusting for covariates. No association was observed for indices *executive* (b = .37, *p* = .062) and *novelty* (b = .45, *p* = .119). Higher education, younger age, and employment were linked to better cognitive function, while preexisting medical conditions did not change the associations. Higher levels of depressive symptomatology were associated with worse cognitive function.

**Conclusions:**

Higher levels of verbal demands at work were associated with better cognitive function for older adults with increased dementia risk. This suggests an advantage for older persons in jobs with high mental demands even after retirement and despite prevalent risk factors. Longitudinal studies are warranted to confirm these results and evaluate the potential of workplaces to prevent cognitive decline through increased mental demands.

## Background

Currently, more than 50 million people worldwide are living with dementia, a number predicted to increase to 152 million until 2050 [1]. In Germany, the corresponding figure is 1.7 million people, with a predicted increase to 2.2 million by 2030 [2]. In the absence of curative treatment options, identifying modifiable risk and protective factors for dementia is a pivotal challenge. Several potentially modifiable factors increasing risk for dementia have been identified, including low education, hearing loss, traumatic brain injury, arterial hypertension, obesity, excessive alcohol consumption (> 21 units per week), diabetes mellitus, depression, physical inactivity, smoking, social isolation, and exposure to air pollution [3].

In addition to these risk factors, a growing number of studies has investigated the relationship between cognitively stimulating workplaces and later-life cognitive functioning [4–7]. Corresponding studies have investigated a variety of concepts of workplace mental demands, including e.g. complexity with data, people or objects, intellectual demands or job control in relation to cognitive function (for a review, please see [8]). Employment constitutes an integral part of most people’s lives, and the average length of occupational history in the European Union is currently 36 years, with a further increase likely in the near future [9]. Moreover, workplace mental demands are experienced at a time in life when a decline in cognitive functioning usually emerges [10]. If mental demands in the workplace contribute to better cognitive functioning in later life, this would indicate a large window of opportunity for prevention of cognitive decline and dementia, especially since modern labor markets increasingly rely on jobs characterized by cognitive rather than physical demands [11].

Protective effects of mental demands on cognition are most often accounted for by the concept of *cognitive reserve*: Cognitively demanding activities and environments are assumed to contribute to an individual’s cognitive reserve, which enables the brain to compensate for neuropathology associated with aging and disease [12, 13]. Another strand of research assumes that high mental demands experienced in, for example, leisure activities or occupations provide opportunities for exercise of higher brain functions, supporting cognitive functioning into older age [14]. Changes in the respective activities and demands over the course of life and, therefore, non-use of cognitive skills and processes result in cognitive decline (use-it-or-lose-it-hypothesis [15]).

Higher levels of mental demands at work were found to be longitudinally associated with better cognitive functioning in old age [6, 16] and a decreased risk for incident dementia [14, 17]. The majority of these studies used data from population-based cohorts. However, a need for studies extending previous findings to different populations has been highlighted [4, 18]. Currently, evidence on associations between mental demands at work in individuals at increased risk for dementia is still scarce. Previous studies were often limited to specific occupational groups like, for example, white-collar workers in the Whitehall II-study [19, 20]. Moreover, only a limited number of studies have investigated associations of mental work demands and cognitive function using data from Germany. Due to demographic changes and an aging population, prolonging working lives constitutes a key priority for German social policy. Therefore, investigating links between work demands and health parameters such as cognitive function in older populations are of valuable interest. Lastly, evidence on potentially modifiable risk factors for dementia has evolved rapidly during the last decade, allowing us to control for various health conditions that have not been addressed in earlier works on workplace mental demands and cognition. The present study therefore aims to investigate the association between mental demands in the workplace and cognitive function in a sample of older individuals at increased risk for dementia, using an objective, comprehensive measure of mental demands experienced in the workplace.

## Methods

### Data

We investigated the association between mental demands at work and cognitive functioning in participants of the *AgeWell.de*-trial. *AgeWell.de* is an ongoing cluster-randomized multi-centric trial investigating the effects of a multi-component lifestyle intervention on cognitive functioning in a sample of older adults (age at baseline: 60–77 years) at increased risk for dementia, according to Cardiovascular Risk Factors, Aging, and Incidence of Dementia (CAIDE)-score [21]. The CAIDE-score covers information on age, education, gender, blood pressure, body mass index, total cholesterol and physical activity, resulting in an additive risk score. Patients with a CAIDE-score ≥ 9 were eligible for participation in *AgeWell.de*. Recruitment took place at five study sites in Germany (Leipzig, Greifswald, Kiel, Munich, and Halle). For a detailed description of the study design and aims, please see [22]. Baseline characteristics of the study sample have been described elsewhere [23]. *AgeWell.de* has been registered in the German Clinical Trials Register (DRKS; ID: DRKS00013555).

The original sample comprised *n* = 1030 participants. Of these, 29 observations were excluded due to missing occupational information (*n* = 16 did not provide occupational information, *n* = 13 in occupations with incomplete Occupational Information Network (O*NET)-data; including *n* = 8: soldiers and other military professions; *n* = 5: taxi drivers/chauffeurs). Four observations were excluded due to incomplete data on cognitive functioning and 56 due to missing information on further covariates, resulting in a final sample of *n* = 941 observations.

### Cognitive functioning

Cognitive functioning was assessed using neuropsychological tests during an interview at the participants’ home. Assessment included the Montreal Cognitive Assessment (MoCA) as a global assessment of cognitive function [24]. The MoCA is a short screening tool to detect cognitive impairment, covering the domains memory, visuospatial abilities, executive function, attention/working memory, language, reasoning, and orientation [24]. Higher values indicate better cognitive performance, the highest possible score being 30 points. The MoCA has shown higher diagnostic accuracy in detecting cognitive deficits and mild cognitive impairment (MCI) than e.g., the Mini Mental State Examination (MMSE [25];). Baseline results revealed a mean MoCA-score of 24.5 points in the *AgeWell.de*-study population, indicating a mildly cognitively impaired sample as to the cut-off suggested by the test developers. Test performance was ≥26 points in 39.4% of participants, indicating unimpaired cognitive function. However, recent studies of older memory clinic outpatients as well as meta-analyses suggest a cut-off of 23/24 points as a more accurate indicator of mild cognitive impairment [25, 26].

### Mental demands at work

Participants provided their current or former main occupation during the baseline interview. This occupational information was then translated into English and respective job titles were matched to an O*NET-code (https://www.onetcodeconnector.org/), using pre-defined criteria such as corresponding task descriptions and responsibilities. O*NET is funded by the US-American Department of Labor/Employment and Training Administration and contains standardized information on a large variety of occupations, including worker abilities and required skills, typical workplace characteristics etc. The database is updated regularly, with information provided by labor market specialists, supervisors, and incumbents of the respective occupation. The current version of the O*NET (version 21.51) comprises 1016 occupational titles, 923 of which with sufficient occupational information on the respective jobs.

As pointed out by Then and colleagues, not all types of mental demands at work show the same associations with cognitive function [5]. Among many specific dimensions of mental demands in the workplace, the dimensions of executive and verbal demands were found to be particularly protective for cognitive functioning [27, 28]. Further, we included the dimension of novelty into our analyses to capture associations of confrontation with new tasks and use of innovative thinking at work with cognition. While experimental studies provide strong evidence for a protective effect of novelty and flexibility on gray matter volume and cognitive function [29, 30] and, conversely, a negative effect of routinization on cognition [31], studies assessing the association of novelty at work and cognitive function are still rare. Certain investigations reported protective effects of workplaces providing a high degree of task switching on cognitive functioning and gray matter volume [32]. Similarly, flexible work demands were found to partially compensate age-related cognitive impairment [33]. Therefore, we followed the approach of Then and colleagues [27] and created three indices of mental demands at work: *Executive*, comprising tasks including independent planning and performance of tasks; *Verbal,* measuring cognitive stimulation of verbal intelligence; *Novelty*, indicating the degree of creativity, innovation, and confrontation with new tasks. A description of the respective indices and included O*NET-variables is provided in Appendix 1. Cronbach’s alpha for the indices *executive* and *verbal* was 0.95 and 0.93, indicating very high internal consistency. The level of internal consistency was acceptable (0.76) for the index *novelty*.

### Other covariates

Gender, age at baseline examination, education, relationship status (married or cohabitating vs. single, divorced, widowed or living apart), employment status (employed vs. retired), diagnoses of diabetes mellitus type 1 or 2, arterial hypertension, history of myocardial infarction, obesity, stroke, total cholesterol (≤/> 6.5 mmol/l), physical activity (at least 2 times per week, at least 30 min.: yes/no), were included as covariates. We further included current depressive symptomatology, as assessed using the Geriatric Depression Scale (GDS) [34]. Education was assessed using the CASMIN-classification (Comparative Analysis of Social Mobility in Industrial Nations [35]), incorporating information on general and vocational education. The general practitioner (GP) provided information on diagnoses of diabetes mellitus, myocardial infarction, stroke, obesity (i.e. body mass index ≥30 kg/m^2^), arterial hypertension and level of total cholesterol to control for established risk factors for cognitive decline and dementia. We identified two individuals who were unemployed at the time of the baseline assessment. The focus of our analyses was on comparing individuals currently working with observations no longer in employment. We therefore decided to include these two observations in the retired-subsample.

### Statistical analyses

Statistical analyses were conducted using Stata 16.0 SE (StataCorp., College Station, TX, USA). We applied generalized linear models (GLM) to address the problem of non-normal distribution of residuals. An alpha level of 0.05 (two-tailed) was chosen to indicate statistical significance. All analyses were run separately for the indices *executive, verbal* and *novelty*. In a first step, we investigated the association of mental demands in the workplace and cognitive functioning, controlling for gender, education and age at baseline (Model 1). Thereupon, we included relationship status, GP diagnoses, physical activity and depressive symptoms into the model (Model 2). Lastly, we investigated potential moderating effects of employment status (employed vs. retired), both independently and in interaction with level of mental demands (employed*index *executive/verbal/novelty*), and controlled for possible interactions of gender with mental demands at work (gender*index *executive/verbal/novelty)* (Model 3). To test robustness of our results, we further assessed associations with mental demands indices and specific cognitive domains. The analyses included measures of working memory, task-switching ability and executive control (assessed using the Trail Making Test B (TMT-B) [36]) and verbal fluency, assessed via both phonemic and semantic fluency (Verbal Fluency Test (VFT) [37]).

## Results

### Sample description

Table 1 describes the total sample. Participants were on average 68.8 years old, with 53.0% being women. 78.6% had already retired, 64.1% were living in a partnership. About half (54.1%) had a medium level of education, with 23.2 and 22.7% reporting low or high levels of education, respectively.

**Table 1:** Participant characteristics (*n* = 941)

Characteristic	Mean (SD)/ %
Age (years; mean, SD)	68.8 (4.9)
Women, %	53.0
*Education (CASMIN; %)*	
Low	23.2
Medium	54.1
High	22.7
*Relationship status, %*	
Married / cohabitating	64.1
Single / divorced / widowed / living apart	35.9
*Employment status, %*	
Employed	21.4
Retired	78.6
MoCA sum score (points; mean, SD)	24.6 (2.9)
*Mental demands at work*	
Index Executive	3.1 (.7)
Index Verbal	3.5 (.6)
Index Novelty	3.5 (.6)
*Diagnoses as reported by the attending GP, %*	
Diabetes mellitus	38.4
History of stroke	4.4
History of myocardial infarction	5.5
Arterial hypertension	87.1
Obesity	54.3
Total cholesterol > 6.5 mmol/l	55.4
Physical activity (≥ 2 times per week, ≥ 30 min)	25.3
Depressive symptoms (GDS; mean, SD)	1.6 (2.0)

Results displayed as means (standard deviation) or percentages, respectively; CASMIN: Comparative Analysis of Social Mobility in Industrial Nations; GDS: Geriatric Depression Scale; GP: general practitioner; MoCA: Montreal Cognitive Assessment; SD: standard deviation

### Multivariate analyses

Tables 2, 3, and 4 report the results of GLMs for the indices *executive, verbal* and *novelty*.

**Table 2:** Association between mental demands at work and cognitive functioning: Index executive

	Model 1			Model 2			Model 3		
	Coeff.	95% CI	p	Coeff.	95% CI	p	Coeff.	95% CI	p
Constant	21.74	20.66; 22.82		22.45	21.17; 23.74		21.08	18.41; 23.75	
Index executive	.24	−.03; .50	.084	.20	−.07; .46	.149	.37	−.02; .75	.062
Age 60–65	Ref.			Ref.			Ref.		
Age 66–70	−.32	−.76; .13	.164	−.30	−.74; .15	.195	−.14	−.60; .33	.563
Age 71–77	**−.98**	−1.42; −.55	<.001	**−.99**	− 1.43; −.56	<.001	**−.80**	− 1.27; −.34	.001
Female gender (ref.: male)	**1.05**	.69; 1.41	<.001	**.95**	.57; 1.32	<.001	1.52	−.06; 3.10	.060
Education low	Ref.			Ref.			Ref.		
Education medium	**.95**	.51; 1.39	<.001	**.97**	.53; 1.42	<.001	**.97**	.53; 1.41	<.001
Education high	**2.26**	1.71; 2.81	<.001	**2.18**	1.63; 2.74	<.001	**2.14**	1.59; 2.69	<.001
Partnership (ref.: single)				−.13	−.52; .25	.503	−.11	−.50; .27	.560
Diabetes mellitus				−.20	−.58; .17	.294	−.18	−.56; .20	.338
Stroke				−.51	−1.37; .35	.245	−.55	−1.41; .31	.209
Myocardial infarction				.21	−.57; .99	.604	.14	−.64; .92	.720
Arterial hypertension				−.24	−.78; .29	.370	−.23	−.76; .30	.402
Obesity				.07	−.31; .45	.720	.10	−.28; .48	.621
Total cholesterol > 6.5 mmol/l				**.38**	.01; .76	.049	.37	−.01; .74	.058
Physical activity				−.19	−.61; .22	.360	−.19	−.61; .22	.364
Depressive symptoms				**−.16**	−.25; −.06	.001	**−.15**	−.24; −.06	.001
Employed (ref.: retired)							1.74	−.15; 3.63	.071
Employed*Index executive							−.39	−.98; .19	.188
Gender (female) *index executive							−.18	−.68; .32	.486
AIC	4.868101			4.863692			4.862841		

AIC: Akaike Information Criterion; CI: confidence interval; Coeff.: coefficient; education assessed according to CASMIN (Comparative Analysis of Social Mobility in Industrial Nations) classification categories low, middle, and high; depressive symptoms assessed using the Geriatric Depression Scale; physical activity: ≥ 2 times per week, ≥ 30 min; diabetes mellitus, stroke, myocardial infarction, arterial hypertension, obesity, total cholesterol as reported by attending GP; significant associations presented in bold type

**Table 3:** Association between mental demands at work and cognitive functioning: Index verbal

	Model 1			Model 2			Model 3		
	Coeff.	95% CI	p	Coeff.	95% CI	p	Coeff.	95% CI	p
Constant	20.88			21.64	20.27; 23.01				
Index verbal	**.50**	.20; .80	.001	**.46**	.15; .76	.003	**.69**	.24; 1.13	.002
Age 60–65	Ref.			Ref.			Ref.		
Age 66–70	−.32	−.77; .12	.156	−.30	−.75; .14	.182	−.15	−.61; .31	.532
Age 71–77	**−1.00**	−1.43; −.57	<.001	**−1.01**	−1.44; −.58	<.001	**−.83**	−1.30; −.37	<.001
Female gender (ref.: male)	**1.01**	.66: 1.37	<.001	**.91**	.54; 1.28	<.001	1.70	−.27; 3.68	.090
Education low	Ref.			Ref.			Ref.		
Education medium	.88	.44; 1.33	<.001	**.91**	.47; 1.36	<.001	**.91**	.47; 1.35	<.001
Education high	2.06	1.50; 2.62	<.001	**2.00**	1.43; 2.56	<.001	**1.97**	1.41; 2.53	<.001
Partnership (ref.: single)				−.15	−.53; .24	.458	−.11	−.49; .27	.565
Diabetes mellitus				−.18	−.56; .19	.344	−.17	−.54; .21	.386
Stroke				−.53	−1.40; .32	.219	−.55	−1.41; .31	.207
Myocardial infarction				.22	−.56; .99	.587	.15	−.62; .93	.689
Arterial hypertension				−.27	−.80; .26	.326	−.23	−.76; .30	.387
Obesity				.06	−.32; .44	.764	.09	−.29; .47	.641
Total cholesterol > 6.5 mmol/l				.37	−.01; .74	.055	.35	−.03; .73	.069
Physical activity				−.19	−.60; .23	.374	−.20	−.61; .22	.355
Depressive symptoms				**−.15**	−.24; −.06	.001	**−.15**	−.24; −.06	.001
Employed (ref.: retired)							**2.77**	.35; 5.18	.025
Employed*Index verbal							−.64	−1.31; .02	.059
Gender (female) *index verbal									.445
AIC	4.860221			4.856579			4.854249		

AIC: Akaike Information Criterion; CI: confidence interval; Coeff.: coefficient; education assessed according to CASMIN (Comparative Analysis of Social Mobility in Industrial Nations) classification categories low, middle, and high; depressive symptoms assessed using the Geriatric Depression Scale; physical activity: ≥ 2 times per week, ≥ 30 min; diabetes mellitus, stroke, myocardial infarction, arterial hypertension, obesity, total cholesterol as reported by attending GP; significant associations presented in bold type

**Table 4:** Association between mental demands at work and cognitive functioning: Index novelty

	Model 1			Model 2			Model 3		
	Coeff.	95% CI	p	Coeff.	95% CI	p	Coeff.	95% CI	p
Constant	21.83	20.45; 23.20		22.54	20.96; 24.11		19.78	15.62; 23.94	
Index novelty	.18	−.15; .51	.293	.14	−.19; .47	.414	.45	−.12; 1.02	.119
Age 60–65	Ref.			Ref.			Ref.		
Age 66–70	−.31	−.76; .13	.169	−.29	−.74; .16	.201	−.15	−.61; .32	.536
Age 71–77	**−.97**	−1.40; −.53	<.001	**−.98**	−1.41; −.55	<.001	**−.80**	−1.27; −.34	.001
Female gender (ref.: male)	**1.04**	.68; 1.41	<.001	**.94**	.56; 1.32	<.001	**2.32**	.02; 4.62	.048
Education low	Ref.			Ref.			Ref.		
Education medium	**.97**	.53; 1.41	<.001	**.99**	.55; 1.43	<.001	**.97**	.53; 1.41	<.001
Education high	**2.32**	1.77; 2.87	<.001	**2.24**	1.68; 2.79	<.001	**2.16**	1.60; 2.72	<.001
Partnership (ref.: single)				−.11	−.50; .27	.569	−.09	−.48; .29	.633
Diabetes mellitus				−.20	−.58; .18	.300	−.18	−.55; .20	.360
Stroke				−.49	−1.35; .37	.266	−.57	−1.43; .29	.196
Myocardial infarction				.23	−.55; 1.01	.571	.16	−.62; .94	.690
Arterial hypertension				−.23	−.76; .31	.402	−.22	−.75; .31	.415
Obesity				.07	−.31; .45	.721	.09	−.29; .47	.648
Total cholesterol > 6.5 mmol/l				**.38**	.01; .76	.046	**.39**	.01; .76	.045
Physical activity				−.20	−.62; .22	.351	−.20	−.61; .22	.351
Depressive symptoms				**−.16**	−.25; −.07	.001	**−.15**	−.24; −.06	.001
Employed (ref.: retired)							1.32	−1.29; 3.93	.323
Employed*Index novelty							−.23	−.97; .51	.547
Gender (female) *index novelty							−.38	−1.03; .26	.243
AIC	4.870115			4.86522			4.864645		

AIC: Akaike Information Criterion; CI: confidence interval; Coeff.: coefficient; education assessed according to CASMIN (Comparative Analysis of Social Mobility in Industrial Nations) classification categories low, middle, and high; depressive symptoms assessed using the Geriatric Depression Scale; physical activity: ≥ 2 times per week, ≥ 30 min; diabetes mellitus, stroke, myocardial infarction, arterial hypertension, obesity, total cholesterol as reported by attending GP; significant associations presented in bold type

Higher levels of index *executive* were not associated with better performance on the MoCA (Table 2). Female gender was associated with higher test scores. Higher age (71–77 years) was associated with lower scores. Medium and high levels of education were linked to better MoCA test scores. Looking at Model 2, higher levels of depressive symptomatology were associated with lower performance in the MoCA, while elevated cholesterol was linked to better test performance. In the final model (Model 3), neither employment status nor the interaction between employment status and index *executive* were associated with performance in the MoCA. The association of index *executive* with MoCA test score did not vary by gender, as expressed by the non-significant interaction term. Elevated cholesterol and female gender were no longer associated with performance in the MocA in the final model.

Higher values of index *verbal* were linked to higher scores in the MoCA (Table 3). Both medium and high levels of education were associated with better test performance, as was female gender. The association between higher values of index *verbal* and performance on the MoCA remained significant when adjusting for covariates. Higher levels of depressive symptoms were linked to lower test performance, while employment was associated with better test performance, compared to retirement. Again, neither the interaction of employment and index *verbal* nor the interaction of index *verbal* and gender explained variation between observations.

No association between values of index *novelty* and MoCA test performance was detected (Table 4). Female gender was linked to better test scores, as were medium and high levels of education. Being 71 years of age or older was linked to lower test scores. After adjusting for covariates, elevated total cholesterol was linked to better test performance, while current depressive symptomatology was associated with lower MoCA-scores. Neither employment status nor the interaction of employment status and novelty explained variation between subjects. Associations between index *novelty* and MoCA test performance did not vary by gender.

The respective models were also applied using the TMT-B and VFT as outcome variables. In the fully adjusted models, higher levels of index *verbal* were linked to better performance on the TMT-B (b = − 18.23; 95% CI: − 27.29; − 9.18; p = <.001), but not in the VFT (*p* = .088). Indices *executive* and *novelty* were not associated with better performance on these tests when adjusting for covariates (index *executive*, TMT-B: *p* = .099; VFT: *p* = .122; index *novelty*, TMT-B: *p* = .243; VFT: *p* = .971).

## Discussion

Our study investigated the association between mental demands at work (i.e.: O*NET-indices *executive, verbal,* and *novelty*) and a global measure of cognitive function in older men and women at increased risk for dementia. Higher levels of the mental demands index *verbal* were associated with better cognitive function after controlling for covariates. These results are in line with other investigations reporting links between higher mental demands in the workplace and cognitive functioning in population-based samples [18, 28, 38, 39]. Corroborating other studies, specifically verbal demands were linked to better performance in tests of cognitive functioning [27, 28, 39]. Regular performance of the respective tasks could serve as training of higher-order cognitive functions, supporting the use-it-or-lose-it-hypothesis. We found no association between mental demands related to innovation, task variation or creativity, as captured by the demands index *novelty,* and cognitive function. As our analyses rely on a sample of older adults at increased risk for dementia, these results indicate that high mental demands targeting verbal intelligence are linked to better cognitive functioning despite prevalent risk factors. Contrary to findings from previous studies [27, 28, 39], higher levels of index *executive,* comprising tasks such as independent planning, scheduling of tasks concerning oneself and others (e.g. teams, subordinates) were not associated with better cognitive function when controlling for covariates. Possible explanations are discussed below.

In contrast to previous research, our study sample included older adults with a high proportion of prevalent risk factors for cognitive decline and dementia. This finding could imply that a certain amount of risk factors for cognitive decline might render associations between better cognitive function in older age and high executive demands in the workplace insignificant. Another possible explanation refers to the interplay of specific dimensions of work, for example the dimensions of demands and possibilities for control, as outlined in the job demand-control-model by Karasek [40]. Certain studies reported that combinations of demands and control (e.g. job strain, defined as high demands and low control, or low demands and low control) are linked to worse cognitive function [41–43].

While executive demands in our study were assessed using objective criteria from an occupational information database, i.e. the O*NET, studies applying the demand-control-framework usually assess subjective perceptions of demands and control experienced at work. The use of objective assessments of workplace demands might have contributed to the non-significant association of executive tasks and cognitive function. We cannot conclude whether the respective job demands were experienced as stressful or challenging, which might influence possible associations with cognitive function. Future studies might address this issue by complementing objective job descriptors with participants’ subjective evaluations of their (former) workplace.

Higher levels of index *novelty* were not associated with global cognitive function. Possibly, occupations characterized by high degrees of novelty exhibit lower levels of job control and require high levels of flexibility, which could be perceived as stressful and, therefore, negatively impact cognitive function. A closer look at our sample revealed that occupations with high levels of index *novelty* included, for example, (preschool) teachers, therapists or engineers, jobs usually characterized by a high degree of responsibility for others and a wide variety of tasks. Drawing on the literature investigating the dimensions of demand and control with respect to cognitive function, possible associations might depend on the dimension of control and possibilities to carry out tasks independently [42, 43].

Higher values of index *verbal* were consistently associated with better cognitive function. This index comprises tasks referring to processing of information and communication with colleagues, customers, or clients. The observed association might point towards the importance of information processing for cognitive function: Certain studies found evidence for decreased dementia risk [5] and slower rates of cognitive decline [45] in people who had been employed in occupations with high levels of information processing. Alternatively, work-related social contacts and interactions might contribute to the observed associations. Index *verbal* addresses job characteristics involving contact with others, such as consulting and advising others and various means of communication, e.g. per telephone or email. This interpretation would underscore findings from other studies, reporting that jobs characterized by high complexity involving people are longitudinally associated with better cognitive function and lower risk for dementia [7]. Still, due to differences in operationalization between the respective concepts, comparisons should be made with caution.

Associations of workplace mental demands index *verbal* and cognitive functioning were attenuated when controlling for education, a finding in line with previous studies (for a review, see [11]). Educational attainment might limit the range of occupations available to an individual [46], or it might have stronger associations with cognition than mental demands encountered in the workplace. However, our results suggest that a small but significant association between mental demands at work and cognitive function prevails even after controlling for education. While educational attainment is usually completed in early adulthood, workplace mental demands are experienced for an extensive time, indicating a potential for preservation of cognitive functioning through the design of mentally demanding workplaces. This is supported by several longitudinal studies reporting the effect of education to decline or even become non-significant after controlling for the influence of occupational complexity, suggesting that educational attainment needs to be transferred to intellectually stimulating working environments to protect against cognitive decline [47, 48]. Certain studies suggest that high mental demands at work particularly benefit those with low levels of education regarding later-life cognitive functioning [49]. Alternatively, baseline intelligence might have contributed to both educational attainment and mental demands experienced in the workplace [49].

The interaction of employment status and index *verbal* was not significant in our full model, implying that the association of higher levels of index *verbal* and cognitive function are observable irrespective of employment/retirement status. Being employed was independently associated with better cognitive function in the analyses of index *verbal.* This finding might indicate support for the use-it-or-lose-it-hypothesis, stating that regular use of skills is protective for cognitive function. Alternatively, this finding could point towards processes of reverse causation, whereas higher cognitive abilities allow for prolonged working lives. However, the proportion of individuals still in employment was rather small in our sample (21.4%), therefore, the respective findings should be interpreted with caution.

Our study extends previous findings on mental demands at work by using data from a sample of older adults from Germany at increased risk for dementia, according to the CAIDE-dementia risk-score [21]. Drawing on the most recent evidence for modifiable risk factors for cognitive decline and dementia [3], we controlled for various health conditions known to affect cognition and dementia risk, which had not been addressed in previous studies on the topic. While the majority of research on mental demands at work and cognition has been conducted in Scandinavian or Anglo-American countries, respective studies with large study samples from Germany are still rare. Our findings, therefore, contribute to the knowledge on cognition and mental demands at work in the specific context of the German population.

## Limitations

Due to the cross-sectional design of the current study, we cannot draw conclusions regarding causality of the observed associations. Individuals with higher baseline cognitive functions or higher cognitive reserve might have had more demanding jobs. Future studies are highly warranted to prospectively assess the association between workplace mental demands and cognition in adults at increased risk for dementia, because longitudinal studies provide less clear evidence than cross-sectional studies of the relationship between mental demands at work and the rate of cognitive decline [18, 50]. It has been suggested that mental demands experienced at work are rather associated with baseline cognitive performance than trajectories of cognitive decline [11, 51, 52]. On the other hand, a study by Rodriguez and colleagues using data from a longitudinal cohort study (age of participants at baseline: ≥ 75 years) found evidence for slower rates of cognitive decline for individuals in jobs with medium levels of mental demands and even more so for individuals with high-level mental demands, indicating a dose-response-relationship observable beyond retirement [45].

While the O*NET-data provide comprehensive and objective measures of workplace mental demands, we cannot rule out the possible influence of subjective evaluations of workplace demands. Individuals employed in the same occupation might rate mental demands experienced at work differently, e.g., due to differences in task preferences. Further, specific tasks and corresponding demands associated with job titles might vary between different employers.

Unfortunately, we were only able to investigate associations with current or former main occupation, regardless of duration of lifetime employment or working hours. A study using data from the Whitehall II cohort found evidence for increased risks of cognitive decline and lower baseline cognitive performance in workers with longer working hours (> 55 h per week [20];). Then and colleagues [5] were able to show that protective effects on cognition depended on duration of exposure for the demands “information processing” and “pattern detection”. Therefore, we cannot rule out whether the detected associations vary between full-time or part-time employment or depend upon duration of lifetime employment history.

The MoCA provides a measure of global cognitive function, therefore, we cannot conclude whether different results would have been observed when using other measures of cognition, e.g. processing speed or visuospatial abilities. However, we conducted supplementary analyses using the TMT-B and the VFT, whereby higher values of index *verbal* were linked to better performance in the TMT-B. Therefore, we are confident that our analyses provide reliable findings.

We used data from a German sample of older men and women, applying a set of workplace characteristics designed for the US-American labor market. Certain differences between the respective job tasks, activities and mental demands might remain between the same occupation in the German and US-American labor market. However, the process of coding occupational titles was subject to strict evaluation, considering matching task descriptions and status of respective jobs to ensure the best possible matching of the participants’ occupational titles with the O*NET-database, hopefully minimizing potential bias.

Lastly, the mean cognitive performance on the MoCA in the *AgeWell.de* study population (24.5 points) indicated that participants were on average slightly cognitively impaired, therefore, our results cannot readily be compared to results from population-based samples.

## Conclusions

We provided evidence for an association between mental demands at work targeting verbal skills and cognitive functioning in a sample of older adults at increased risk for dementia after adjusting for sociodemographic and health-related confounders known to affect dementia risk. These findings extend existing knowledge on the association of mental demands and cognition, which so far mainly comprises samples from population-based studies or specific occupational groups. Corroborating previous studies, our findings suggest that not all but specific kinds of mental demands experienced in the workplace are linked to better cognitive function [18].

Our findings suggest an advantage of men and women with jobs characterized by high levels of verbal demands regarding cognitive function, observable even after exiting the labor market and in a population with slightly impaired cognitive status. Focusing on clearly defined measures of cognitive function, our study found evidence for associations of verbal demands with better cognitive function, suggesting that verbal demands explain differences in cognitive function in older adults even when a certain decline of cognitive function is visible. However, longitudinal investigations are warranted to expand our knowledge on the relative importance of mental demands at work and their interplay with other known risk factors for cognitive decline and dementia. Future studies should further investigate the potential of other, nonwork environments in providing the respective demands and possible associations with cognitive function to maintain respective benefits in older age.

## Data Availability

The data presented in this study are available from the corresponding author upon reasonable request.

## Appendix 1 Index of mental demands derived from the O*NET-database

*Index executive (Cronbach’s alpha: 0.95)*

**Table:**

4.A.2.b.4	Developing Objectives and Strategies (importance)
4.A.2.b.4	Developing Objectives and Strategies (level)
4.A.2.b.5	Scheduling Work and Activities (importance)
4.A.2.b.5	Scheduling Work and Activities (level)
4.A.4.a.7	Resolving Conflicts and Negotiating with Others (importance)
4.A.4.a.7	Resolving Conflicts and Negotiating with Others (level)
4.A.4.b.1	Coordinating the Work and Activities of Others (importance)
4.A.4.b.1	Coordinating the Work and Activities of Others (level)
4.A.4.b.4	Guiding, Directing, and Motivating Subordinates (importance)
4.A.4.b.4	Guiding, Directing, and Motivating Subordinates (level)

*Index verbal (Cronbach’s alpha: 0.93)*

**Table:**

4.A.1.a.1	Getting Information (importance)
4.A.2.a.3	Evaluating Information (importance)
4.A.2.a.3	Evaluating Information (level)
4.A.2.b.3	Updating and Using Relevant Knowledge (importance)
4.A.2.b.3	Updating and Using Relevant Knowledge (level)
4.A.4.a.1	Interpreting the Meaning of Information (importance)
4.A.4.a.1	Interpreting the Meaning of Information (level)
4.A.4.b.6	Providing Consultation and Advice (importance)
4.A.4.b.6	Providing Consultation and Advice (level)
4.C.1.a.2.f	Telephone
4.C.1.a.2.h	Email
4.C.1.a.2.j	Letters

*Index novelty (Cronbach’s alpha: 0.76)*

**Table:**

1.B.1.f	Conventional (reversed)
1.B.2.f	Independence
1.C.4.c	Adaptability/Flexibility
1.C.7.a	Innovation
4.A.2.b.2	Thinking creatively (level)
4.A.2.b.2	Thinking creatively (importance)
4.C.3.b.7	Importance of Repeating same Task (reversed)

## Abbreviations

AIC: Akaike Information Criterion
BMBF: German Federal Ministry for Education and Research
CAIDE-score: Cardiovascular Risk Factors, Aging, and Incidence of Dementia Risk Score
CASMIN: Comparative Analysis of Social Mobility in Industrial Nations
CI: confidence interval
Coeff.: coefficient
DRKS: German Clinical Trials Register
GDS: Geriatric Depression Scale
GLM: generalized linear model
GP: general practitioner
MCI: mild cognitive impairment
MMSE: Mini Mental State Examination
MoCA: Montreal Cognitive Assessment
O*NET: Occupational Information Network
SD: standard deviation
TMT-B: Trail Making Test B
VFT: Verbal Fluency Test
